# Visceral Leishmaniasis Treatment, Italy

**DOI:** 10.3201/eid0912.030178

**Published:** 2003-12

**Authors:** Luigi Gradoni, Marina Gramiccia, Aldo Scalone

**Affiliations:** * Istituto Superiore di Sanità, Rome, Italy

**Keywords:** Visceral leishmaniasis, drug treatment, pentavalent antimony, amphotericin B, Italy

## Abstract

First-line drug treatment was recorded in 573 immunocompetent patients with visceral leishmaniasis in Italy. In the past 12 years, the proportion of antimonial treatments decreased from 100% to 2.8%, while the proportion of amphotericin B treatments increased from 0% to 97.2%. The countrywide change in therapy is a response to both disease reemergence and increasing antimonial failure.

Zoonotic visceral leishmaniasis is a life-threatening disease caused by the multiplication of the protozoan parasite *Leishmania infantum* in the phagocytes of the reticuloendothelial system. Infections are widespread in the Mediterranean subregion, where the parasite is transmitted in summer by the bites of phlebotomine sand flies, and canids serve as reservoir hosts (*1*).

In the first half of the 20th century, visceral leishmaniasis was a typical infantile syndrome in Italy with high incidence in southern regions and islands. After World War II, the incidence dropped to 10 to 20 cases per year for 4 decades; the disease reemerged with approximately 200 cases in 2000 and 2001 (Figure 1). This trend can be explained by the following: 1) the appearance of cases in immunocompetent adults that might be attributable to a general decrease in acquired immunity after the reduction of the phlebotomine-vector populations, determined by the massive antimosquito insecticide campaigns for malaria eradication 50 years ago (*2*); 2) the spreading of the disease from traditional areas of transmission to new stable foci in central and northern regions of Italy, as evidenced by recent colonization of these areas by sand flies and by increased *Leishmania* diffusion and prevalence among the canine reservoir (*3*); and 3) the occurrence of *Leishmania* infections in immunosuppressed persons, such as those co-infected with HIV (*4*). Incidence of visceral leishmaniasis in these patients, however, has recently decreased after the introduction of highly active antiretroviral therapy therapy (Figure 1) (*5*).

**Figure 1:**
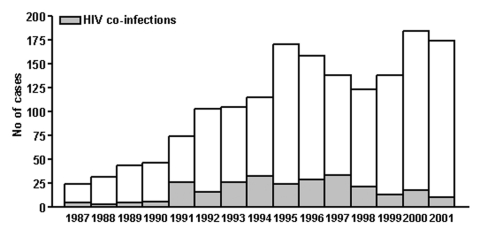
Reemergence of zoonotic visceral leishmaniasis in Italy: human cases recorded from 1987 through 2001 by passive repots and active surveillance.

Since the 1940s through 1990, meglumine antimoniate has been the only first-line drug for visceral leishmaniasis treatment in Italy (*6*). From 1991 through 1994, a total of 90 patients of all ages, representing one third of all immunocompetent visceral leishmaniasis case-patients reported in Italy during that period, were enrolled in clinical trials of liposomal amphotericin B (L-AmB), which led to a novel, safe, short course of visceral leishmaniasis treatment as an alternative to meglumine antimoniate (*7*,*8*). In the same period, other lipid-associated AmB drugs were registered in Italy for the treatment of fungal infections, i.e., AmB colloidal dispersion (ABCD) and AmB lipid complex (ABLC). Because no official policy exists for visceral leishmaniasis therapy in Italy (physicians can prescribe any registered drug under their own responsibility) and information on drug regimens used is not included in visceral leishmaniasis case reports, we aimed to assess whether changes have occurred, and to what extent, in first-line drug regimens adopted in Italy after lipid-associated AmB was introduced into clinical practice.

## The Study

A retrospective analysis was performed on data collected at the Unit of Protozoology of Istituto Superiore di Sanità, Rome, the main reference center for visceral leishmaniasis surveillance in Italy. Diagnosis of visceral leishmaniasis in patients with clinically suspected cases was routinely performed on serum and bone marrow aspirate samples sent by hospitals, mainly from pediatrics, internal medicine, and infectious diseases wards, from throughout the country. If visceral leishmaniasis was confirmed, relevant information on patients was recorded, which included drug regimens used and posttherapy results. Two datasets were analyzed: the first included information from patients in whom leishmaniasis was diagnosed from 1986 to 1990, i.e., before the mass enrollment of patients in the aforementioned study on L-AmB; the second from patients in whom leishmaniasis was diagnosed from 1995 to 2001, i.e., after that study. Immunosuppressed patients (e.g., HIV co-infected persons or transplant recipients), who usually respond poorly to antileishmanial treatments, were not included in our analysis. Fisher exact test was used for comparisons.

For the 1986–1990 period, we recorded treatments used for 40 patients in 22 hospitals, representing 29.2% of 137 immunocompetent persons with visceral leishmaniasis. Fourteen (35.0%) were children <14 years of age. As expected, all patients were treated with meglumine antimoniate, given at the standard dose of 20 mg pentavalent antimony (Sb^v^)/kg/day for 3 to 4 weeks (*6*), either alone (37 patients) or in combination with allopurinol at the daily dose of 15 mg/kg (3 patients). Two patients treated with meglumine antimoniate alone (5.4%) had a visceral leishmaniasis relapse within 6 months from treatment and have been retreated successfully with meglumine antimoniate in combination with allopurinol.

**Figure 2:**
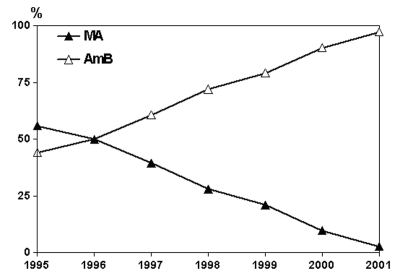
Annual proportion of immunocompetent patients with visceral leishmaniasis treated with meglumine antimoniate (MA) or amphotericin B (AmB) in the period 1995–2001.

For the 1995–2001 period, we recorded treatment information for 533 patients, representing a large proportion (56.4%; annual range 43.3% to 69.1%) of 945 immunocompetent visceral leishmaniasis patients. About half were children (267; annual range in proportion 42.1% to 64.8%). Every year, patients were referred by 19 to 42 hospitals, with a range of 1 to 30 patients per hospital. Drug regimens recorded are shown in the Table and summarized in Figure 2. Meglumine antimoniate was the first-line drug used in 158 patients (29.6%) at the Sb^v^ dosages noted previously; 6 also received allopurinol (the drug combination was used until 1997). The proportion of meglumine antimoniate-treated patients has steadily decreased from 55.9% in 1995 to 2.8% in 2001. AmB drugs have been the only alternative drugs used in the remaining 375 patients (70.4%). Of those patients, L-AmB accounted for most regimens (348, 92.8%); this drug was administered to both children and adults at the standard dose of 3 mg/kg/day for 5 consecutive days plus an additional 3 mg/kg dose on day 10 (*7*). Slight variations from this regimen (e.g., 3 mg/kg/day for 7–10 consecutive days) were recorded in some case-patients. ABCD and ABLC were given only to adult patients (21 and 3 cases, respectively) both at the dosage of 2 mg/kg/day for 7 days. Thus, lipid-associated AmB was used to treat 372 patients (69.8%). Finally, three adult patients were treated with the conventional AmB deoxycholate formulation (dosages unreported). The proportion of patients treated with any AmB-based drugs increased from 44.1% in 1995 to 97.2% in 2001.

**Table:** First-line drugs used for treatment of visceral leishmaniasis in 533 immunocompetent patients in Italy and drug treatment failures recorded^a^

Y	MA (%)	L-AmB	ABCD	ABLC	dAmB	Any AmB drugs (%)
1995	57 (55.9) 3 R	45 1 R	0	0	0	45 (44.1) 1 R
1996	35 (50.0) 2 R	35 1 R	0	0	0	35 (50.0) 1 R
1997	26 (39.4) 3 U, 1 R	34 2 R	5	0	1	40 (60.6) 2 R
1998	14 (28.0) 1 R	32 1 R	4	0	0	36 (72.0) 1 R
1999	13 (21.0) 1 U, 1 R	45 2 R	2	1	1	49 (79.0) 2 R
2000	11 (9.8) 1 U, 3 R	89 1 U, 1 R	10 1 U	1 1 R	1	101 (90.2) 2 U, 2 R
2001	2 (2.8)	68 1 R	0	1	0	69 (97.2) 1 R
Total	158 (29.6) 5 U, 11 R	348 1 U, 9 R	21 1 U	3 1 R	3	375 (70.4) 2 U, 10 R

^a^MA, meglumine antimoniate; L-AmB, liposomal amphotericin B; ABCD, amphotericin B colloidal dispersion; ABLC, amphotericin B lipid complex; dAmB, amphotericin B desoxycholate; U, unresponsiveness and/or acute toxicity; R, relapse.

We recorded the failure of drug therapy in 16 (10.1%) of 158 patients treated with meglumine antimoniate, equally distributed in children and adults and with no association with particular geographic location. Five patients showed primary unresponsiveness or experienced acute toxicity, which required suspension from treatment, while 11 patients who responded initially to treatment had a relapse after a variable period of time (range 3–11 months). All patients were successfully retreated with the standard L-AmB regimen. Altogether, the rate of meglumine antimoniate failures recorded in 1995 to 2001 did not differ significantly from that of the failures in 1986 to 1990. However, the rate significantly increased in recent years, from 3 (5.3%) of 57 in 1995 to 4 (36.4%) of 11 in 2000 (p = 0.01). Drug treatment was unsuccessful in 12 (3.2%) of 375 AmB-treated patients, in 2 patients the infection was unresponsive, and 10 patients had a relapse at 3 to 10 months. This rate was significantly lower than the meglumine antimoniate failure rate (p = 0.002). Drug treatment failed in 8 of 10 L-AmB–treated children from different geographic locations. All AmB treatment failures but one (retreated with meglumine antimoniate) were successfully retreated with a high-dose L-AmB regimen of 3 mg/kg/day for 10 consecutive days.

## Conclusions

A range of treatment options exists in visceral leishmaniasis, which include two pentavalent antimonials (meglumine antimoniate and sodium stibogluconate), four formulations of AmB, aminosidine (paromomycin), pentamidine, and the new oral agent miltefosine (*9*,*10*). For the clinician, the choice of treatment depends on several factors, such as the clinical features of the disease, as well as drug safety, efficacy, and cost. In the absence of any official drug policy for visceral leishmaniasis in Italy and in consideration of the large sample of patients surveyed, our study may be the first observational investigation on visceral leishmaniasis therapy at the national level from 1986 through 2001.

Results have shown a countrywide change in therapy over the period considered. Even though the change was relatively gradual over a 16-year period, a traditionally effective drug (meglumine antimoniate) has been almost fully replaced by a new compound, L-AmB, in an epidemiologic context of disease reemergence. Possible explanations for this change include the following: 1) mild or severe adverse reactions (e.g., pancreatitis, cardiac abnormalities) are commonly seen in meglumine antimoniate–treated patients, especially in adults, when the recommended dosages are increased even slightly. A recent investigation in Sicily showed that the antimony-associated death rate was 7% among HIV-negative adults with or without underlying diseases (*11*). On the other hand, the toxicity of lipid-associated AmB drugs, especially L-AmB, was negligible in all categories of patients at the dosages used for visceral leishmaniasis therapy (*9*). 2) The efficacy of meglumine antimoniate for the treatment of Mediterranean visceral leishmaniasis has been high (approximately 95%) for >50 years. However, in the past few years, meglumine antimoniate treatment failures have increased in visceral leishmaniasis patients from southern Europe. This treatment failure could be attributable to the widespread use of meglumine antimoniate in treating infected dogs, which may have caused the spread of *L. infantum* strains less susceptible to antimony (*12*–*14*). Efficacy of AmB drugs is very high and, so far, decreased *Leishmania* susceptibility to this compound (AmB is rarely used in veterinary practice) has not been indicated. 3) Although L-AmB and other lipidic formulations of AmB are 30- to 50-fold more expensive than meglumine antimoniate for visceral leishmaniasis therapy at the dosages reported above, in Western countries most of the costs of treating visceral leishmaniasis are inpatient hospitalization expenses rather than drug costs. Therefore, short courses of 6 to 7 days, as required for L-AmB, ABCD, or ABLC (*9*), are highly cost-effective if compared with 21- to 28-day courses needed for meglumine antimoniate treatment.

## References

[1] Desjeux P. The increase in risk factors for leishmaniasis worldwide. Trans R Soc Trop Med Hyg. 2001;95:239–43. 10.1016/S0035-9203(01)90223-8 11490989

[2] Mansueto S, Barba G, Cerrito B, Farinella E, Orsinis S, Di Rosa S. Visceral leishmaniasis of adults in Sicily: a truce interrupted? Trans R Soc Trop Med Hyg. 1987;81:161–2. 10.1016/0035-9203(87)90314-2 3445306

[3] Gradoni L. Epizootiology of canine leishmaniasis in southern Europe. In: R. Killick-Kendrick, editor. Canine leishmaniasis: an update. Proceedings of the Canine Leishmaniasis Forum, Barcelona, Spain. Wiesbaden, Germany: Hoechst Roussel Vet; 1999. p. 32–9.

[4] Gradoni L, Scalone A, Gramiccia M. Epidemiological surveillance of leishmaniasis in HIV-1-infected individuals in Italy. AIDS. 1996;10:785–91. 10.1097/00002030-199606001-00014 8805871

[5] del Giudice P, Mary-Krause M, Pradier C, Grabar S, Dellamonica P, Marty P, Impact of highly active antiretroviral therapy on the incidence of visceral leishmaniasis in a French cohort of patients infected with human immunodeficiency virus. J Infect Dis. 2002;186:1366–70. 10.1086/344325 12402211

[6] Gradoni L, Bryceson A, Desjeux P. Treatment of Mediterranean visceral leishmaniasis. Bull World Health Organ. 1995;73:191–7. 7743590 PMC2486749

[7] Davidson RN, di Martino L, Gradoni L, Giacchino R, Gaeta GB, Pempinello R, Short course treatment of visceral leishmaniasis with liposomal amphotericin B (AmBisome). Clin Infect Dis. 1996;22:938–43. 8783690 10.1093/clinids/22.6.938

[8] Meyerhoff AUS. Food and Drug Administration approval of AmBisome (lipsomal amphotericin B) for treatment of visceral leishmaniasis. Clin Infect Dis. 1999;28:42–8. 10.1086/515085 10028069

[9] Davidson RN. Practical guide for the treatment of leishmaniasis. Drugs. 1998;56:1009–18. 10.2165/00003495-199856060-00005 9878989

[10] Jha TK, Sundar S, Thakur CP, Bachmann P, Karbwang J, Fisher C, Miltefosine, an oral agent, for the treatment of Indian visceral leishmaniasis. N Engl J Med. 1999;341:1795–800. 10.1056/NEJM199912093412403 10588964

[11] Cascio A, Gradoni L, Scarlata F, Gramiccia M, Giordano S, Russo R, Epidemiologic surveillance of visceral leishmaniasis in Sicily, Italy. Am J Trop Med Hyg. 1997;57:75–8. 9242323 10.4269/ajtmh.1997.57.75

[12] Gramiccia M, Gradoni L, Orsini S. Decreased sensitivity to meglumine antimoniate (Glucantime) of *Leishmania infantum* isolated from dogs after several courses of drug treatment. Ann Trop Med Parasitol. 1992;86:613–20. 1304703 10.1080/00034983.1992.11812717

[13] Faraut-Gambarelli F, Piarroux R, Deniau M, Giusiano B, Marty P, Michel G, In vitro and in vivo resistance of *Leishmania infantum* to meglumine antimoniate: a study of 37 strains collected from patients with visceral leishmaniasis. Antimicrob Agents Chemother. 1997;41:827–30. 9087498 10.1128/aac.41.4.827 PMC163803

[14] Carrió J, Portús M. In vitro susceptibility to pentavalent antimony in *Leishmania infantum* strains is not modified during in vitro or in vivo passages but is modified after host treatment with meglumine antimoniate. BMC Pharmacol. 2002;2:11. 10.1186/1471-2210-2-11 12019027 PMC113748

